# Optimizing miRNA transfection for screening in precision cut lung slices

**DOI:** 10.1152/ajplung.00138.2024

**Published:** 2024-09-10

**Authors:** Joanna Nowakowska, Nika Gvazava, Wojciech Langwiński, Kamil Ziarniak, Iran Augusto N. da Silva, John Stegmayr, Darcy E. Wagner, Aleksandra Szczepankiewicz

**Affiliations:** ^1^Molecular and Cell Biology Unit, Department of Pediatric Pulmonology, Allergy and Clinical Immunology, Poznan University of Medical Sciences, Poznan, Poland; ^2^Doctoral School, Poznan University of Medical Sciences, Poznan, Poland; ^3^Lung Bioengineering and Regeneration, Department of Experimental Medical Sciences, Faculty of Medicine, Lund University, Lund, Sweden; ^4^Lund Stem Cell Center, Faculty of Medicine, Lund University, Lund, Sweden; ^5^Wallenberg Center for Molecular Medicine, Faculty of Medicine, Lund University, Lund, Sweden; ^6^NanoLund, Lund University, Lund, Sweden; ^7^Meakins-Christie Laboratories, The Research Institute of the McGill University Health Centre, Montreal, Quebec, Canada; ^8^Department of Biomedical Engineering, McGill University, Montreal, Quebec, Canada

**Keywords:** miRNA, precision cut lung slices, transfection

## Abstract

Precision cut lung slices (PCLS) are complex three-dimensional (3-D) lung tissue models, which preserve the native microenvironment, including cell diversity and cell-matrix interactions. They are an innovative ex vivo platform that allows studying disease as well as the effects of therapeutic agents or regulatory molecules [e.g., microRNA (miRNA)]. The aim of our study was to develop a protocol to transfect PCLS with miRNA using lipid nanoparticles (LNPs) to enable higher throughput screening of miRNA, obviating the need for custom stabilization and internalization approaches. PCLS of 4 mm diameter were generated using agarose-filled rodent lungs and a vibratome. TYE665-labeled scrambled miRNA was used to evaluate transfection efficacy of six different commercially available LNPs. Transfection efficacy was visualized using live high-content fluorescence microscopy, followed by higher-resolution confocal fluorescence microscopy in fixed PCLS. Metabolic activity and cellular damage were assessed using water-soluble tetrazolium salt (WST-1) and lactate dehydrogenase (LDH) release. Using a live staining kit containing a cell membrane impermeant nuclear dye, RedDot2, we established that cellular membranes in PCLS are permeable in the initial 24 h of slicing but diminished thereafter. Therefore, all transfection experiments occurred at least 24 h after slicing. All six commercially available LNPs enabled transfection without inducing significant cytotoxicity or impaired metabolic function. However, RNAiMAX and INTERFERin led to increases in transfection efficacy as compared with other LNPs, with detection possible as low as 25 nM. Therefore, LNP-based transfection of miRNA is possible and can be visualized in live or fixed PCLS, enabling future higher throughput studies using diverse miRNAs.

**NEW & NOTEWORTHY** RNA-based therapeutics hold significant promise for disease treatment; however, limited research exists on miRNA transfection specifically within PCLS. miRNA transfection has thus far required custom functionalization for stabilization and internalization. We aimed to optimize a transfection protocol for rapid screening approaches of miRNA sequences. We show that transfecting miRNA in PCLS is possible using lipid nanoparticles. In addition, we show that 25 nM of TYE665-miRNA is sufficient for detection in a high-content imaging system.

## Graphical abstract

**Figure:**
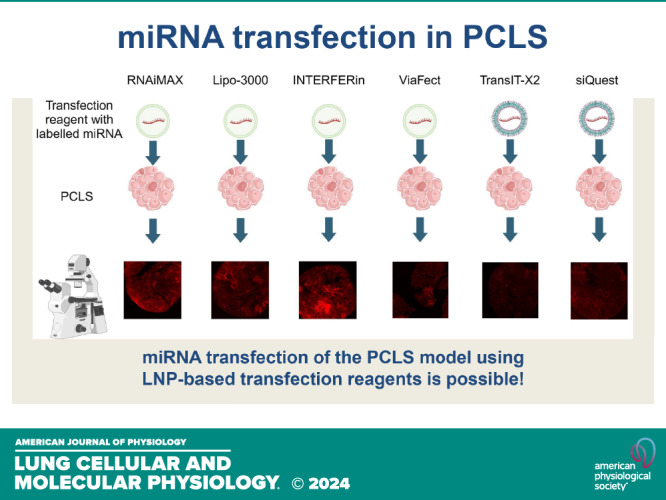

## INTRODUCTION

Respiratory diseases such as chronic obstructive pulmonary disease (COPD), lower respiratory infections, and lung cancer are among the top causes of death globally and are known to be caused by a combination of environmental injuries and genetic susceptibility (1). They remain without cure and are predicted to further increase due to the close relationship of damaging agents with factors associated with climate change and globalization. There is thus a pressing need for new experimental approaches to accelerate the development of new therapies for respiratory disease.

The lungs are made up of over 40 different cell types that closely interact with each other. Recent large-scale single-cell transcriptomic approaches have highlighted the relationship between multiple cell types in the onset and progression of disease (2). For this reason, in vitro culture systems involving only one or even a few types of cells will incompletely reflect the high complexity of interactions between different cell types, which is critical for identifying potential therapies.

Precision cut lung slices (PCLS) are an alternative culture method, which was first described several decades ago for use in toxicity testing but has recently seen renewed interest due to the fact that human tissue can be used and because they permit the study of multiple cell populations in their native spatial arrangement, including the extracellular matrix. There are several devices that can be used to generate PCLS, and they can be generated to have standard cross-sectional areas (3), which also makes them more amenable to screening approaches (4). PCLS can reduce the number of animals used in experiments or even replace animals altogether, which is in line with the application of the “Four Rs” rule in animal welfare (replacement, reduction, refinement, and responsibility). The increased throughput of experiments possible with PCLS makes them ideal as a screening platform to perform studies evaluating the effect of potential therapies across multiple conditions (i.e., timepoints, concentrations, and types of therapy).

MicroRNAs (miRNAs) are small (∼22 nucleotides), single-stranded, noncoding RNA molecules, which simultaneously control post-transcriptional regulation of hundreds of genes (5). They are involved in many fundamental cellular functions, and changes in their expression have increasingly been shown to play a pivotal role in many diseases (6), including respiratory diseases such as asthma (7), COPD (8), lung cancer (9), and idiopathic pulmonary fibrosis (10). Therefore, miRNAs have both diagnostic and therapeutic potential with several therapies already at the stage of human clinical trials for nonrespiratory indications, including, e.g., heart failure, diabetes, and solid tumors (11). Thus, there is high interest in miRNAs research, especially when considering their pleiotropic actions in cells and changes in their expression in the course of disease.

However, numerous miRNA targets have been identified as differentially regulated in respiratory diseases through the use of short RNA sequencing approaches of normal and diseased lung tissue/cells as well as by computationally predicting differentially regulated miRNAs from mRNA transcriptomic approaches (12, 13). The diversity of miRNAs identified to date in different pulmonary diseases makes screening of specific miRNA sequences challenging. Although treatment of individual cell types is possible, miRNAs are known to target multiple transcripts simultaneously and thus they have diverse effects depending on the specific cell in which they are expressed (or delivered therapeutically) (11). Thus, their study in complex cellular models containing as many cell types as possible in their native spatial orientation is particularly important. Recent work has shown that miRNA mimics with enhanced chemical stability via peptide conjugation show promise in exerting antifibrotic activities in human PCLS (14, 15), but such modifications need to be individually optimized and assessed for each candidate miRNA as some of them may alter biological activity. Combined, these challenges make rapid screening of candidate miRNAs challenging. Thus, we aimed to identify suitable commercially available miRNA transfection reagents in PCLS that are effective but do not induce significant cellular toxicity. Furthermore, we sought to confirm whether an imaging based-approach with single-cell resolution could be used to confirm cellular uptake in PCLS. Such a platform would allow for future usage in miRNA screening platforms using PCLS and high-content imaging.

## MATERIALS AND METHODS

### Animals and PCLS Generation

Lung tissue was obtained from healthy C57BL/6J mice (*n* = 4) and Wistar male rats (*n* = 2). In line with the 4-R principles, we utilized excess tissue from animals scheduled for euthanasia for tissue harvest in other studies. Mice were anesthetized via an intraperitoneal injection of sodium pentobarbital (40–50 mg/kg) prior to organ harvest. Rats were euthanized by decapitation. PCLS of 300 µm thickness were prepared using a vibratome (Campden Instruments, 7000smz-2), with the exception that 1.5% agarose was used, and cultured as previously described using DMEM-F12 medium (supplemented with 1% penicillin/streptomycin and 0.1% fetal bovine serum) in a standard humidified incubator (37°C, 5% CO_2_) (16). A 4-mm biopsy punch was used to generate slices of normalized volume for culture in 100 µL of complete medium in 96-well plates with daily medium changes (one 4 mm diameter slice in each well with 100 μL medium) (3). Ciliary beating was routinely assessed pretransfection as a surrogate for PCLS viability (16, 17) using a ×20 ELWD objective in a Nikon Eclipse Ts2R microscope equipped with a highspeed camera (Imaging Source, DFK 33UX264).

### Analysis of Membrane Permeabilization and Apoptosis in Freshly Cut and Cultured PCLS

PCLS punches were cultured in 100 μL of complete medium in a 4-well culture-insert plate (ibidi, Germany) and stained at different timepoints, as described in the respective figure legend, using the NucView 488 and RedDot2 Apoptosis and Necrosis kit (Biotium, California) according to the manufacturer’s protocol and visualized in living PCLS using a Leica STELLARIS 5 confocal microscope (Leica, Germany). Identical laser settings were used for all experiments and across all timepoints. Settings were selected so that no signal was observed in nonstained, time-matched PCLS and to avoid saturation in stained samples, using FITC and Cy5 laser settings of 15% and 6% excitation intensity and 107 and 29 gain, respectively. Z-stacks of ∼140 μm total were acquired with a z-step of 5 μm. Also, 150 μM hydrogen peroxide treatment was used as a positive control for evaluating the stability of the dyes over time under conditions to induce cellular membrane damage and apoptosis (18); a z-step size of 2.4 μm was used in these experiments. FITC and Cy5 channels were collected sequentially to avoid spectral overlap. A multicolor, spectral lookup table (LUT) was applied to single-plane images using the Leica LAS X Software to aid in the visualization of different pixel intensities. All raw imaging data are available in the BioImage Archive of the EMBL (S-BIAD1284).

### Transfection

Transfection was performed according to the manufacturer’s recommendation for each transfection reagent, with their respective abbreviation used throughout the manuscript [lipid-based: RNAiMAX, Lipofectamine3000 (Lipo3000), INTERFERin, ViaFect, and nonliposomal polymeric systems TransIT-X2 and TransIT-siQuest (siQuest)]. Transfection mixtures were prepared in Opti-Mem (Thermo Fisher Scientific, Massachusetts) at 0, 25, 50, or 100 nM. A complete culture medium was added to each well to obtain 100 μL total volume. Each experiment included both untreated samples (marked as CTRL) and mock control samples (transfection reagent without miRNA). A custom synthesized scrambled miRNA molecule conjugated to a far-red dye, TYE-665, at the 3′ end, was used for all experiments (Qiagen, Germany) as a negative control. For transfection with an inhibitor targeting a relevant mRNA, we used rno-miR-223-3p unlabeled inhibitor at a concentration of 50 nM using INTERFERin as the transfection reagent and TYE665-labeled scrambled miRNA as the control sequence.

PCLS were fixed for 30 min using 10% neutral buffered formalin solution (Sigma, HT501128) at room temperature, then replaced with sterile, 1X phosphate-buffered saline (SH30256, HyClone Cytiva) and stored at 4°C until imaging.

### miRNA and RNA Analysis

For nucleic acid extraction 48 h after the transfection, each PCLS was placed in 200 µL of Qiazol (Qiagen) and stored at −80°C after isolation according to the manufacturer’s protocol (one PCLS in 200 µL of Qiazol as one experimental replicate). RNA and miRNA samples from PCLS transfected with rno-miR-223-3p inhibitor and negative control (scrambled miRNA inhibitor) were reverse transcribed using a GoScript Reverse Transcription kit (Promega, Wisconsin) for target mRNA and a TaqMan Advanced miRNA cDNA Synthesis kit for rno-miR-223-3p (Thermo Fisher Scientific, Massachusetts).

Using the miRTarBase tool (19), we selected the experimentally validated target for rno-mir-223-3p, insulin-like growth factor receptor 1 (*Igf1r*). Relative expression analysis was done using quantitative reverse transcription PCR (qRT-PCR). GoTaq qPCR Master Mix (Promega, Wisconsin) was used with specific *Igf1R* primers (F: 5′- CCAACGGATTGATTCTAATG-3′; R: 5′- CACAGGATCTGTCCACGAC-3′) and *Gapdh* as a reference gene (F: 5′- CACTCCCTCAAGATTGTCAGCAA-3′; R: 5′- GGCATGGACTGTGGTCATGA-3′). We assessed rno-miR-223-3p expression with TaqMan Advanced miRNA assay (Thermo Fisher Scientific, Massachusetts), using miR-26a as an endogenous control.

### Water-Soluble Tetrazolium Salt Assay

Water-soluble tetrazolium salt assay (WST-1 Assay) (Sigma-Aldrich, Missouri) was performed in triplicate and used to assess proliferation and viability of individual punches, as we have previously described (3). Absorbance was measured at 440 nm using a multimode microplate reader (Cytation5, BioTek,) against a background control (DMEM F-12 medium) as blank and reference wavelength of 620 nm.

### Lactate Dehydrogenase Assay

Lactate dehydrogenase activity (Cytotoxicity detection kit LDH, Sigma-Aldrich, Missouri) was assessed daily in secreted supernatants, according to the manufacturer’s protocol and as we previously described (20). In the initial screening experiments, supernatants from eight technical replicates were pooled at each time point prior to LDH analysis. Supernatants were diluted in DMEM-F12 medium prior to use (60:40 vol/vol). Absorbance at 490 nm was measured using a microplate reader (Cytation5, BioTek), with 620 nm as a reference, against a background control (DMEM-F12 medium). At each time point, separate PCLS treated with 2% Triton X-100 solution for 30 min, to induce total cell lysis and maximal LDH release, was used and normalized in the same way as the other data presented on each graph, as follows: For all screening experiments in mice, relative absorbance at each time point posttransfection was normalized to the sum of LDH released (i.e., sum of relative absorbance) in the 4 days prior to transfection, as noted in the respective figure legend. The following formula was used:

Rel ARel AD4=Rel Aposttransfection, time pointRel Apretransfection

where “A” represents absorbance and “Rel A” represents relative absorbance as defined by (A_490nm_–A_620nm_). For rat PCLS screening experiments, relative absorbance at each time point was normalized to the relative absorbance only on *day 3* after slicing (i.e., pretransfection). In experiments where rno-miR-223-3p and mock inhibitor were transfected, data are presented as relative absorbance at each time point and for individual PCLS.

### Live High-Content Widefield Fluorescence Imaging and Conventional Inverted Fluorescence Microscopy to Assess Transfection Efficacy

Murine PCLS were imaged live after transfection using ×4 and ×20 objectives in a high-content imaging system equipped with a widefield camera and GFP and CY5 filter cubes (Cytation5, BioTek) and rat PCLS using a conventional inverted fluorescence microscope (Nikon Ts2R). At the beginning of each imaging experiment, a nontransfected control sample was used to establish the microscope settings for transfected samples using the CY5 filter cube. For all imaging experiments, LED intensity, camera gain, and image acquisition time were set in the nontransfected control so that there was little to no fluorescence detected in the channel of interest and that pixel intensity saturation was limited in Cy5, where TYE665 is detected (21). This approach was taken to ensure that any and all fluorescence captured in experimental groups was due to transfection procedures utilizing TYE665 fluorophore. As we were utilizing a high-content imaging system, autofocusing in our three-dimensional (3-D) model was not feasible and thus we captured all CY5 images at the same z-height. Therefore, in parallel to the CY5 images, tissue autofluorescence of PCLS was captured using the GFP filter cube to ensure that images acquired in the CY5 channel corresponded to PCLS tissue (which can float within an individual well). Settings were then kept the same while all other conditions were imaged. Settings are described in the corresponding figure legends. Bright-field images were acquired in parallel for rat PCLS using an inverted microscope (Nikon Ts2R). Fluorescent images were deconvolved using standard processing parameters in Gen5 Imaging software. Fluorescent intensity was measured in ImageJ using distinct regions, as indicated in each figure legend.

### Confocal Microscopy to Assess Transfection Efficacy

Fixed PCLS were imaged in a Zeiss 780 Confocal Laser-Scanning Inverted Microscope using secure-seal spacers, 9 mm diameter, 0.12 mm deep (Thermo Fisher Scientific, Massachusetts) with ×4, ×10, ×20, and ×40 objectives using standard fluorescence laser settings: Cy5, FITC, and DAPI. Bright-field images of PCLS were acquired in parallel for anatomical referencing independent of autofluorescence.

### Quantification of Apoptosis and Cellular Membrane Damage

Confocal images were analyzed using the in-built Analyze Particles function in ImageJ software. Thresholds were set individually for each channel and maintained consistently across the quantification of all images for all PCLS and conditions. To ensure that all quantification occurred within well-focused regions of PCLS, we manually selected a thickness of ∼50 µm that was within the interior of each PCLS. Thereafter, every other plane was selected for quantification (to ensure that the same cell was not counted twice as our z-step was 5 μm). This corresponded to five planes per PCLS at each timepoint. The number of features with cross-sectional areas between 25 and 85 µm^2^ was quantified for all conditions (corresponding to a nuclear size of ∼4.5 to 10.5 µm diameter to cover the range of nuclear diameters determined in distal murine lung tissue (6 to 7 µm) in a previous stereology-based study) (22). Additional analysis parameters such as cross-sectional area, circularity, and solidity were also evaluated.

### Statistics

All graphs and statistical analyses were performed with GraphPad Prism (ver 10.1.2) using one- or two-way ANOVA or Student’s *t* test, as indicated in the figure legends. In all analyses, *P* < 0.05 was considered significant.

## RESULTS

### Establishment of a Suitable Time Window for miRNA Transfection

**Figure 1.**
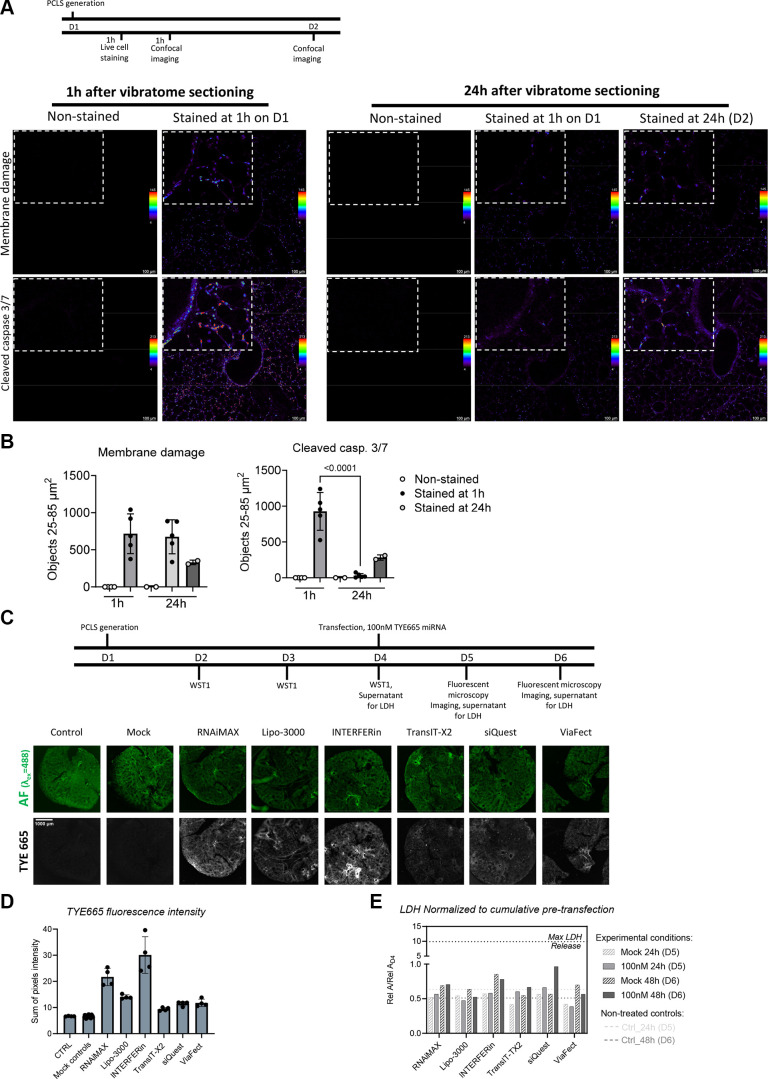
Establishment of a suitable time window for miRNA transfection and screening of transfection reagents in murine PCLS. *A*: experimental overview and single, representative z-plane acquired with confocal microscopy and stained live immediately or 24 h after vibratome slicing; unstained controls; pseudocolored with a multicolor LUT to represent pixel intensity (*n* = 2 male mice with 2–5 PCLS per condition). *B*: quantification of objects corresponding to murine nuclei to assess membrane damage (RedDot2 nuclear stain) and cleaved caspase 3/7 staining (NucView 488 nuclear stain) immediately or 24 h after vibratome sectioning; *P* value < 0.05 considered significant; one-way ANOVA with Šidák correction for multiple comparisons. *C*: experimental overview (*n* = 2 male mice used in the experiment). Representative (8 PCLS used for each experimental group) high-content fluorescent microscopy images 48 h posttransfection, ×4 objective, TYE665 dye (imaged with Cy5 filter settings), and cellular/tissue outline imaged using autofluorescence with GFP filter settings [imaging settings for each filter cube (LED intensity, integration time, and gain): Cy5 (6, 6 ms, and 24) and GFP (10, 30 ms, and 24)]. *D*: sum of TYE665 fluorescence intensity (*n* = 8 regions); no statistical significance found using two-way ANOVA for all comparisons. *E*: LDH release posttransfection from supernatants pooled from eight PCLS, normalized to cumulative pretransfection release. LDH, lactate dehydrogenase; LUT, lookup table; miRNA, microRNA; PCLS, precision cut lung slices.

As our primary goal was to identify transfection agents capable of protecting miRNA from extracellular degradation and permitting intracellular delivery in PCLS, we first sought to identify a suitable time window for transfection. The majority of papers utilizing cultured PCLS describe an overnight or 24 h time period to allow the PCLS to recover following sectioning. In particular, cellular membrane damage due to the slicing procedure itself could be a major confounding factor for studies assessing the efficiency of delivery agents, such as with transfection reagents. Therefore, we sought to characterize cell membrane permeability in murine PCLS after slicing using a live staining setup containing a cell membrane impermeant nuclear dye, RedDot2, and NucView 488, a caspase 3/7 sensitive nuclear dye. We first performed live staining immediately after PCLS generation (i.e., no rest period) followed by live confocal imaging. We observed clear evidence of cell membrane permeability, as assessed by visible RedDot2 nuclear staining (Fig. 1*A*, Supplemental Fig. S1). In addition, we observed NucView 488 nuclear staining, indicating activation of caspase3/7 immediately after slicing. However, after a further 24 h of incubation, both RedDot2 and NucView 488 staining were subjectively reduced. NucView 488 nuclear staining was significantly decreased (*P* < 0.0001) between the first and second days of culture while decreases observed in RedDot2 were not statistically significant (Fig. 1*B*). On the other hand, PCLS that were allowed to rest and stained 24 h after slicing had reduced NucView 488 and RedDot2 staining as compared with those stained immediately after slicing (Fig. 1*B*).

To confirm that the loss of fluorescence we observed, especially with the NucView 488 dye, was not due to photobleaching or dye instabilities, we treated PCLS with 150 μM hydrogen peroxide immediately after slicing in parallel experiments to induce sustained cell damage. The live staining dyes were added on *day 2* (i.e., 24 h after slicing), and PCLS were imaged immediately thereafter as well as after an additional 24 h (i.e., 48 h after slicing). We found that fluorescence was retained over time, with subjective increases in NucView 488 staining and sustained RedDot2 staining (Supplemental Fig. S2), indicating that both dyes were stable. Furthermore, the size distribution for the particles we quantified remained constant across all individual PCLS punches and timepoints (Supplemental Fig. S3) and is in line with previous literature regarding nuclear size in the murine lung (22). However, we observed a loss of circularity and solidity after 24 h of culture for both NucView 488 and RedDot2, irrespective of when staining was performed (Supplemental Fig. S3). This is in line with previous reports of loss of circularity and solidity in nuclei during apoptosis (23), which we have previously observed with culture time in PCLS using histological approaches (16). Taken together, this confirms that the dyes are specific and stable under our culture conditions and that vibratome slicing induces membrane permeability and apoptosis, which is present during the initial 24 h but begins to subside thereafter.

### Screening of the Transfection Reagents

After establishing a minimum time window with which to conduct PCLS transfection, we proceeded to screen 6 commercially available transfection reagents. Four of them are lipid-based nanoparticles (LNPs) (RNAiMAX, Lipofectamine3000, INTERFERin, and ViaFect) and two are nonliposomal polymeric systems (TransIT-X2 and TransIT-siQuest). We chose to perform our initial screen at 4 days after slicing due to our own data and as this time frame has been suggested in other studies to be outside the peak of inflammation seen in PCLS supernatants (24). We routinely confirmed tissue viability by assessing ciliary beating in the airways of PCLS prior to transfection (Supplemental Video S1). To allow for real-time visualization of cellular uptake in PCLS, we used a custom miRNA tagged with TYE665, a far-red dye at 100 nM to mimic a higher range of commonly used in vitro concentrations of miRNA, which have been shown to exert biologic effects without inducing cytotoxicity in two-dimensional (2-D) cell culture.

We detected intracellular red fluorescence well-above levels seen in control conditions at both 24 and 48 h in PCLS treated with TYE665-miRNA, but its intensity varied depending on the transfection reagent(Fig. 1, *C* and *D*). None of the transfection reagents, with or without miRNA, induced major amounts of LDH release when compared with the nontransfected controls and all were orders of magnitude less than the maximal theoretical LDH release (Fig. 1*E*, dotted line), indicating negligible release of LDH with all transfection reagents. As the highest fluorescence was observed for RNAiMAX, Lipofectamine3000, and INTERFERin, these transfection reagents were chosen for further screens.

### Evaluation of Reduced miRNA Concentrations Using the Three Most Effective Transfection Reagents

**Figure 2.**
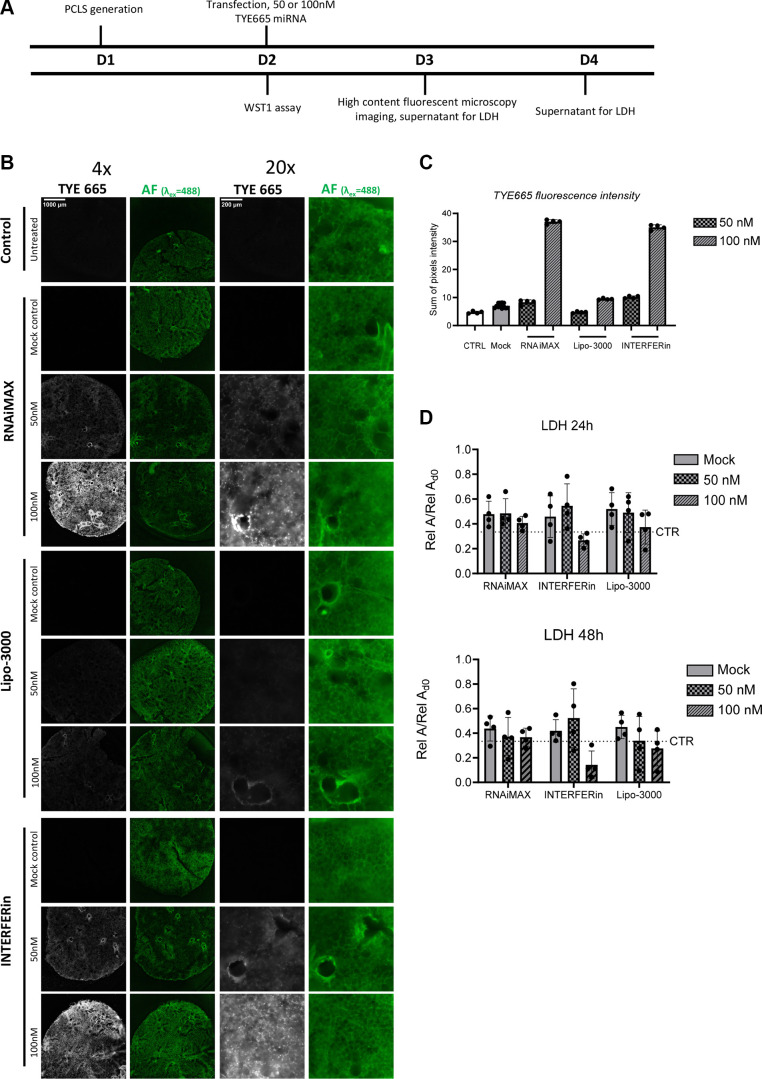
Optimization of miRNA concentration for the three most effective transfection reagents. *A*: experimental timeline for murine PCLS (*n* = 2 male mice used in the experiment). *B*: representative (4 PCLS used for each experimental group) fluorescent microscopy images 24 h posttransfection, ×4 and ×20 objectives, TYE665 (imaged using Cy5 filter settings), and cellular/tissue outline imaged using autofluorescence using GFP filter settings [imaging settings for each filter cube (LED intensity, integration time, and gain) at ×4—Cy5 (6, 6 ms, and 24) and GFP (10, 30 ms, and 24); ×20—Cy5 (6, 8 ms, and 18), and GFP (10, 15 ms, and 24)]. *C*: sum of TYE665 fluorescence intensity (4 regions). *D*: LDH release posttransfection, normalized to LDH release pretransfection; dotted line with the abbreviation “CTR” represents the normalized mean value of the controls (i.e., nontransfected) at each time point; no statistical significance was found with two-way ANOVA. LDH, lactate dehydrogenase; miRNA, microRNA; PCLS, precision cut lung slices.

**Figure 3.**
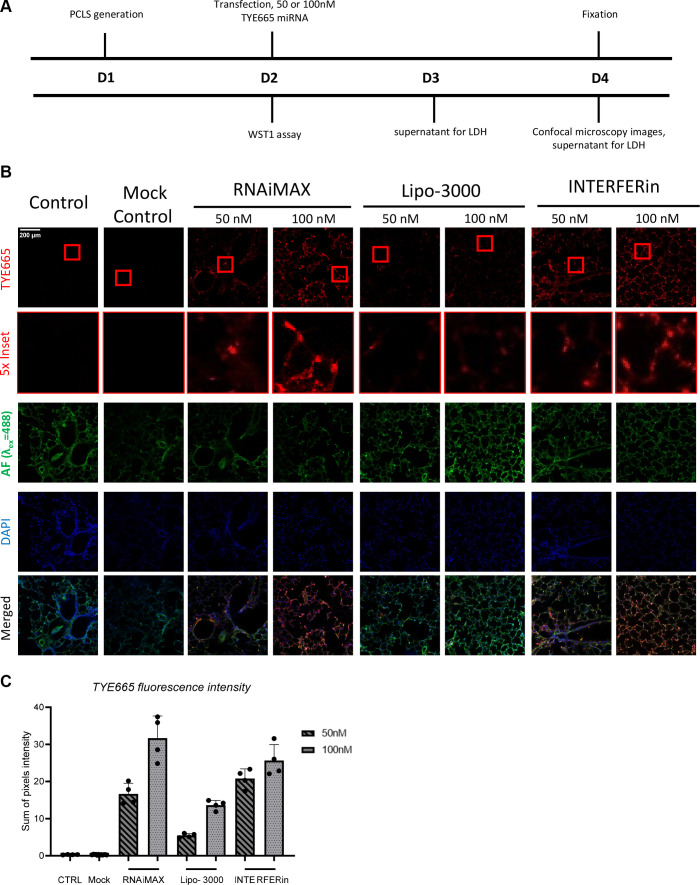
Confocal microscopy of fixed PCLS for the three most effective transfection reagents. *A*: experimental timeline for murine PCLS (*n* = 2 male mice used in the experiment). *B*: representative (1 PCLS from each experimental group) confocal microscopy images of individual channels and merged PCLS fixed 48 h posttransfection, ×20 objective, TYE665 visualized with Cy5 (laser: 5.0, gain: 751), tissue autofluorescence visualized with FITC (laser: 14.0, gain: 800), DAPI (laser: 8.0, gain: 700). *C*: sum of TYE665 fluorescence intensity (*n* = 4 regions). PCLS, precision cut lung slices.

Next, we tested two concentrations of TYE665-labeled miRNA (100 and 50 nM). We observed concentration-dependent amounts of red fluorescence for all transfected samples at 24 h posttransfection (Fig. 2, *A*–*C*). Importantly, 50 nM TYE665-miRNA could be visualized in live samples using high-content widefield epifluorescence microscopy. In line with our previous results (Fig. 1), we did not observe any major LDH release in our secondary screen at either 50 nM or 100 nM or with any transfection agent in comparison with nontransfected controls (Fig. 2*D*). Although conventional epifluorescence microscopy is widely available and permits rapid imaging, it has limited spatial resolution as compared with confocal imaging, even when post-image processing such as deconvolution is used. Therefore, we fixed PCLS after live imaging for use with higher-resolution imaging (confocal microscopy). Similar to our analysis with live PCLS in conventional widefield epifluorescent imaging, we observed concentration-dependent fluorescent intensity with all transfection reagents (Fig. 3, *A–C*).

### Proof of Principle for Performing a miRNA Transfection Screen from a Single Animal

Based on the previous results, we selected RNAiMAX and INTERFERin transfection reagents for further analysis due to the fact that they did not induce noticeable cytotoxicity and resulted in higher fluorescent intensities, which we attributed to higher transfection efficiency. In these experiments, we used 50 and 25 nM miRNA to test the lower limits of concentration, which could be detectable. Furthermore, we extended our work to PCLS from rat lungs as more PCLS can be generated from a single animal to accommodate all endpoints and for screening approaches.

**Figure 4.**
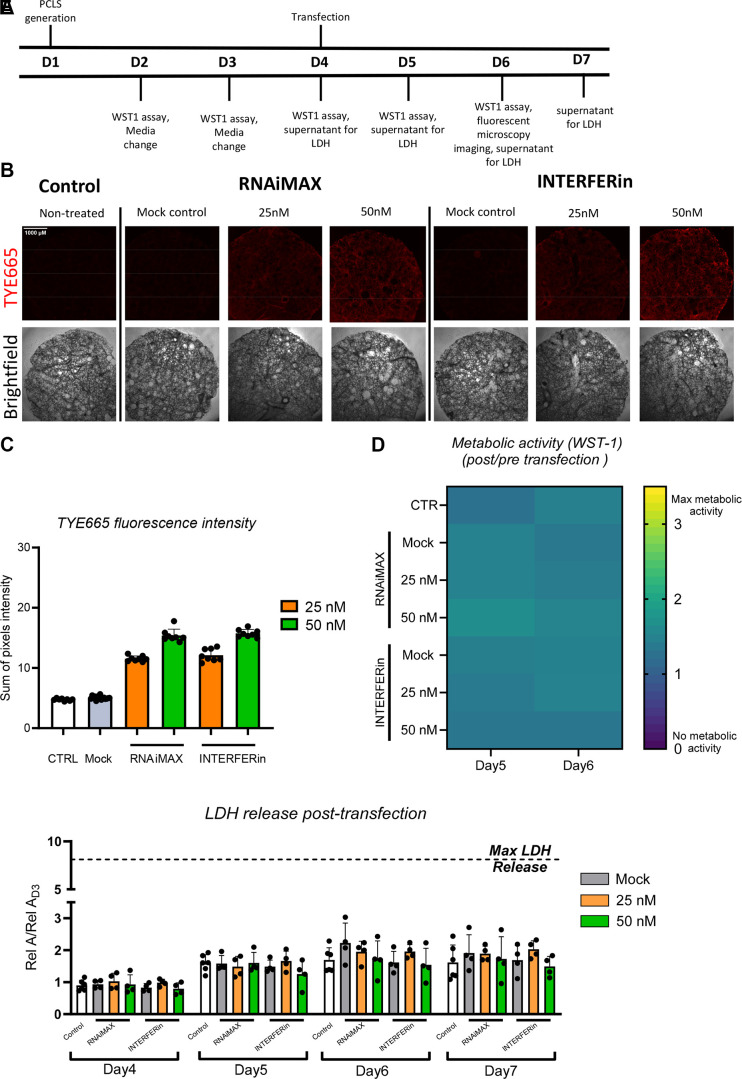
TYE665-miRNA can be detected in rat PCLS using 25 nM. *A*: experimental timeline for rat PCLS (*n* = 1 male rat used in the experiment). *B*: representative (4 PCLS used for each experimental group) bright and widefield fluorescent microscopy 48 h posttransfection, ×4 objective, TYE665 dye (laser: 15, exposure time: 300 ms). *C*: sum of TYE665 fluorescence intensity (8 regions). *D*: metabolic activity (WST-1), heatmap represents absorbance values of individual PCLS. *E*: LDH release on *days 4–7* and normalized to *day 3* media change; each dot represents an individual PCLS (4–6 technical replicates); no statistical significance was found with two-way ANOVA test. LDH, lactate dehydrogenase; miRNA, microRNA; PCLS, precision cut lung slices; WST-1, water-soluble tetrazolium salt.

At both 24 and 48 h after transfection, we detected red fluorescence in all transfected samples. Transfection with 25 nM miRNA was sufficient for the detection of red fluorescence with both RNAiMAX and INTERFERin, which showed similar concentration-dependent fluorescence intensities, indicating similar transfection efficiency (Fig. 4, *A–C*). Neither transfection reagent caused noticeable changes in metabolic activity (Fig. 4*D*) nor significant increases in LDH release as compared with nontransfected controls, confirming that both reagents do not induce any significant cell damage and that PCLS retain good cell viability posttransfection (Fig. 4*E*). In addition, for the assessment of PCLS function, we confirmed the presence of ciliary beating pre- and posttransfection (24 and 48 h) (Supplemental Video S2), indicating retention of tissue-level function posttransfection.

### Transfection with rno-miR-223-3p

**Figure 5.**
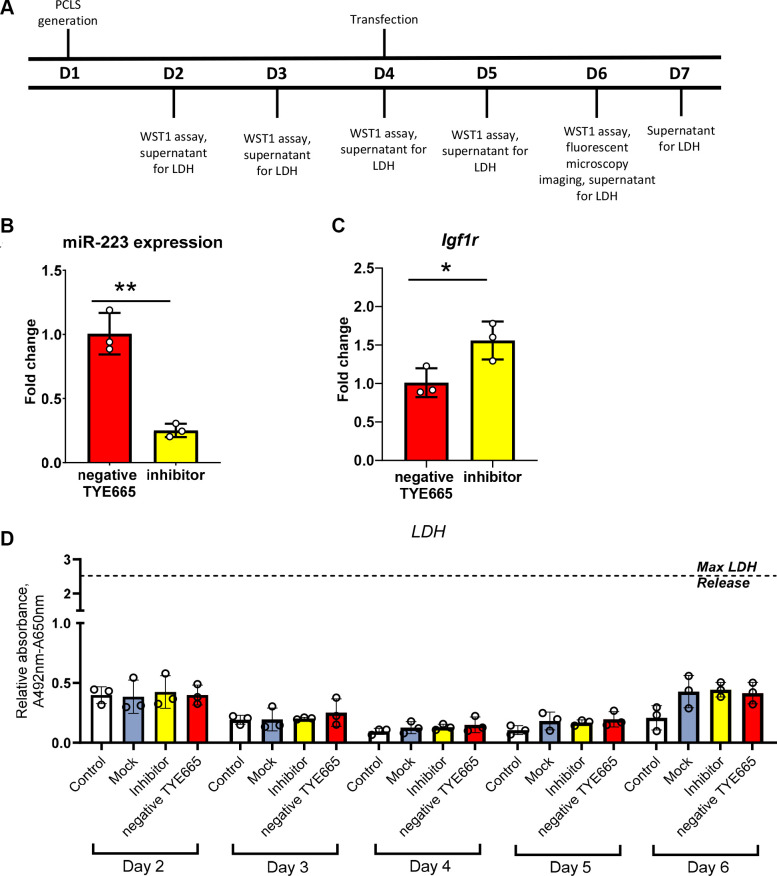
Transfection with rno-miR-223-3p inhibits miR-223-3p levels and corresponds to increases in a known downstream gene, *Igfr1*. *A*: experimental timeline for rat PCLS (*n* = 2 male rats used in the experiment). *B*: qPCR analysis of rno-miR-223-3p expression (*P* = 0.0015), each dot represents an individual PCLS (RNA isolated from a single PCLS; 3 experimental replicates). *C*: qPCR analysis of *Igf1r* expression (*P* = 0.0374), each dot represents an individual PCLS (3 experimental replicates). *D*: LDH release on *days 2–6*; each dot represents an individual PCLS (3 experimental replicates); statistical significance in *B* and *C* was tested using Student’s *t* test with significance considered *P* < 0.05; no statistical significance was found in *D* with two-way ANOVA test. LDH, lactate dehydrogenase; PCLS, precision cut lung slices. *P < 0.05, **P < 0.005.

To assess whether miRNA transfection with our optimized method results in effective knockdown of the target, we performed an experiment using INTERFERin and an antagonist for miR-223, rno-miR-223-3p because we previously identified it as a target of interest in asthmatic rat lungs where it was upregulated (25). In line with our previous experiments, we found that transfection with rno-miR-223-3p inhibitor was effective, with significantly decreased miR-223 in comparison with the negative control (TYE665-labeled scrambled miRNA) (*P* = 0.0015) (Fig. 5*B*). Next, we assessed downstream efficacy by assessed *Igf1r* expression, which has previously been experimentally determined to be targeted by miR-223 (26). Similarly to previous work and as predicted, we found that *Igf1R* expression was significantly increased (*P* = 0.037) in PCLS transfected with rno-miR-223-3p inhibitor in comparison with the negative control (Fig. 5*C*). As we have already shown in the previous experiments, neither the transfection reagent nor the miRNA inhibitor caused significant increases in LDH concentrations compared with any of the controls: untransfected PCLS, mock control, and scrambled miRNA inhibitor (Fig. 5*D*).

## DISCUSSION

Despite the potential for RNA-based therapeutics, there have been limited reports of miRNA transfection in PCLS previously and none that aimed to optimize a protocol for screening approaches. Previous work in RNA inhibition has been carried out with siRNA transfection of organotypic slices (27, 28), including lung, but miRNA requires different transfection reagents. A few studies have recently attempted to transfect PCLS with miRNA but utilized custom-generated miRNAs conjugated to peptides for stability. Although detailed methodological details were not available for both studies, one of the studies performed miRNA transfection using a peptide nucleic acid within the first 24 h after slicing (14). Our own data indicate that nonspecific uptake can occur in PCLS due to membrane permeability occurring due to the slicing procedure. Although inhibition of target mRNA was confirmed in that study, nonspecific uptake may overestimate potential therapeutic effects in vivo. Therefore, we sought to identify a potential experimental time window for which damage due to membrane permeability is minimized to limit nonspecific uptake. This is particularly important for future studies using targeted nanoparticles. We found that membrane damage from the initial slicing was greatly reduced after the first 24 h and thus PCLS were allowed to rest for at least 24 h prior to all transfection experiments. In addition, we demonstrated that transfection can occur at later timepoints (e.g., 72 h after slicing), which is particularly important for PCLS models that induce disease ex vivo over several days in PCLS derived from healthy animals or humans.

We chose to screen commercially available reagents of two different transfection reagent classes: lipid nanoparticles (LNPs) and nonlipid-based polymeric nanoparticles. Although none of the transfection reagents showed toxicity in PCLS, we found that LNPs outperformed polymeric formulations with regard to transfection efficiency. Both LNPs (RNAiMAX and INTERFERin) demonstrated reproducible efficacy in the transfection of PCLS with miRNA across multiple species and screening attempts without significantly affecting the viability of PCLS. LNPs are increasingly recognized as strong candidate delivery vehicles for miRNA-based therapeutics due to their ability to stabilize nucleotides and additionally that targeted moieties can be added to them for cell-specific delivery. For screening approaches, they offer a further benefit in that they allow rapid screening of multiple unmodified miRNAs.

As our analyses showed, the transfection with a specific miRNA inhibitor (rno-miR-223-3p) significantly affected the expression of a target for the miRNA (*Igfr1*) as well as miRNA itself, thus confirming the possibility to obtain effective knockdown with our method. Although we only assessed the feasibility for miRNA in the present study, our high-content imaging approach may be feasible for use in assessing LNP formulations for the delivery of mRNA in PCLS. One of the current bottlenecks in optimizing LNPs for in vivo usage is the high number of formulations, which need to be tested and known differences between in vitro and in vivo transfection efficacies (29, 30). PCLS offer a unique opportunity to test LNP formulations in a more in vivo like setting comprised of diverse cells. mRNA encoding fluorescent or luminescent proteins could be used to demonstrate efficient delivery as well as protection of the nucleotides through endolysosomal escape to enable protein translation.

One of the limitations of the study is that our analyses were only conducted on rodent tissues. It is certainly worth extending our analyses with human PCLS in the future. Furthermore, although we utilized ciliary beating for functional assessment of maintenance of tissue viability (16, 17), future studies should incorporate additional functional analyses such as assessment of airway contraction, Ca^2+^ flux under confocal microscopy, or maintenance of epithelial ion channels (17, 31). Despite these limitations, our works demonstrate that miRNA transfection of the PCLS model using LNP-based transfection reagents is feasible, which opens up a number of new possibilities both in miRNA research and testing other nucleotides with potential therapeutic applications.

## DATA AVAILABILITY

All raw and processed data will be made available upon reasonable request to one of the corresponding authors. All raw imaging data for visualization of apoptosis and cell membrane damage are available in the BioImage Archive of the EMBL (S-BIAD1284).

## SUPPLEMENTAL MATERIAL

10.6084/m9.figshare.26396233 Supplemental Video S1: https://doi.org/10.6084/m9.figshare.26396233.

10.6084/m9.figshare.26396236 Supplemental Video S2: https://doi.org/10.6084/m9.figshare.26396236.

10.6084/m9.figshare.26396239 Supplemental Figs. S1–S3: https://doi.org/10.6084/m9.figshare.26396239.

## GRANTS

This work was supported by Preludium Bis, National Science Center, Grant no. UMO-2020/39/O/NZ5/01804 (to A.S.). This project has received funding from the European Research Council (ERC) under the European Union’s Horizon 2020 research and innovation program (Grant Agreement No. 805361; to D.E.W.). This work was also supported by a SciLife Lab Grant under the COVID-19 pandemic program KAW 2020.0182 (to D.E.W.) and the Swedish Research Council 2018–02352 (to D.E.W.). This research was undertaken, in part, thanks to funding from the Canada Excellence Research Chairs Program (to D.E.W.; CERC-2022-00013).

## DISCLOSURES

No conflicts of interest, financial or otherwise, are declared by the authors.

## AUTHOR CONTRIBUTIONS

J.N., N.G., W.L., J.S., D.E.W., and A.S. conceived and designed research; J.N., N.G., W.L., K.Z., I.A.N.S., and D.E.W. performed experiments; J.N., N.G., D.E.W., and A.S. analyzed data; J.N., N.G., W.L., D.E.W., and A.S. interpreted results of experiments; J.N., N.G., D.E.W., and A.S. prepared figures; J.N. drafted manuscript; N.G., D.E.W., and A.S. edited and revised manuscript; J.N., N.G., W.L., K.Z., I.A.N.S., J.S., D.E.W., and A.S. approved final version of manuscript.
